# Clinical implementation and evaluation of the Acuros dose calculation algorithm

**DOI:** 10.1002/acm2.12149

**Published:** 2017-08-20

**Authors:** Chenyu Yan, Anthony G Combine, Greg Bednarz, Ronald J Lalonde, Bin Hu, Kathy Dickens, Raymond Wynn, Daniel C Pavord, M Saiful Huq

**Affiliations:** ^1^ Department of Radiation Oncology UPMC Cancer Centers Pittsburgh PA USA; ^2^ Department of Radiation Oncology Loyola University Health System Stritch School of Medicine Loyola University Chicago IL USA; ^3^ Health Quest Poughkeepsie NY USA

**Keywords:** Acuros XB, dose calculation algorithm

## Abstract

**Purpose:**

The main aim of this study is to validate the Acuros XB dose calculation algorithm for a Varian Clinac iX linac in our clinics, and subsequently compare it with the wildely used AAA algorithm.

**Methods and materials:**

The source models for both Acuros XB and AAA were configured by importing the same measured beam data into Eclipse treatment planning system. Both algorithms were validated by comparing calculated dose with measured dose on a homogeneous water phantom for field sizes ranging from 6 cm × 6 cm to 40 cm × 40 cm. Central axis and off‐axis points with different depths were chosen for the comparison. In addition, the accuracy of Acuros was evaluated for wedge fields with wedge angles from 15 to 60°. Similarly, variable field sizes for an inhomogeneous phantom were chosen to validate the Acuros algorithm. In addition, doses calculated by Acuros and AAA at the center of lung equivalent tissue from three different VMAT plans were compared to the ion chamber measured doses in QUASAR phantom, and the calculated dose distributions by the two algorithms and their differences on patients were compared. Computation time on VMAT plans was also evaluated for Acuros and AAA. Differences between dose‐to‐water (calculated by AAA and Acuros XB) and dose‐to‐medium (calculated by Acuros XB) on patient plans were compared and evaluated.

**Results:**

For open 6 MV photon beams on the homogeneous water phantom, both Acuros XB and AAA calculations were within 1% of measurements. For 23 MV photon beams, the calculated doses were within 1.5% of measured doses for Acuros XB and 2% for AAA. Testing on the inhomogeneous phantom demonstrated that AAA overestimated doses by up to 8.96% at a point close to lung/solid water interface, while Acuros XB reduced that to 1.64%. The test on QUASAR phantom showed that Acuros achieved better agreement in lung equivalent tissue while AAA underestimated dose for all VMAT plans by up to 2.7%. Acuros XB computation time was about three times faster than AAA for VMAT plans, and computation time for other plans will be discussed at the end. Maximum difference between dose calculated by AAA and dose‐to‐medium by Acuros XB (Acuros_D_m,m_) was 4.3% on patient plans at the isocenter, and maximum difference between D_100_ calculated by AAA and by Acuros_D_m,m_ was 11.3%. When calculating the maximum dose to spinal cord on patient plans, differences between dose calculated by AAA and Acuros_D_m,m_ were more than 3%.

**Conclusion:**

Compared with AAA, Acuros XB improves accuracy in the presence of inhomogeneity, and also significantly reduces computation time for VMAT plans. Dose differences between AAA and Acuros_D_w,m_ were generally less than the dose differences between AAA and Acuros_D_m,m_. Clinical practitioners should consider making Acuros XB available in clinics, however, further investigation and clarification is needed about which dose reporting mode (dose‐to‐water or dose‐to‐medium) should be used in clinics.

## 1 INTRODUCTION

Intensity‐modulated radiation therapy (IMRT) and volumetric‐modulated arc therapy (VMAT) can produce highly conformal radiation dose distributions and enhance treatment localization, but these complex treatment techniques also place higher demands on dose calculation algorithms in terms of both accuracy and computation speed.^1^, ^2^ With the increasing popularity of IMRT and VMAT techniques in clinics, accuracy in treatment planning systems (TPSs) has always been a concern in modern radiotherapy. To address that concern, analytical anisotropic algorithm (AAA) was implemented in the Eclipse (Varian Medical Systems) treatment planning system to replace the pencil beam (PBS) for the calculation of dose distributions for photon beams. The first characterization of the AAA algorithm in water was published by Fogliata et al.^3^ in their investigation of the configuration module of the AAA algorithm, they compared dose calculated by AAA with measurements and reported an accuracy of 1%–2% for output factors of open and wedged beams, respectively, a 1%, 1 mm average accuracy in the calculated depth dose curves and an accuracy within 1% for the central region of the profiles. Esch et al.^4^ reported that AAA improves the accuracy of dose calculations compared to PBS and can achieve 5% agreement with measurements in thoracic phantom.

Even though with the significant improvement, AAA still lacks the accuracy of Monte‐Carlo dose calculation algorithm which is often accepted as the golden standard. Over the past years, it has become a common belief that precise dose calculation will necessitate the use of Monte‐Carlo methods to take correctly into account the electron transport governing the dose deposition process. However, Monte‐Carlo methods are presently still too time consuming to be used in routine clinical environments. Hence, the impetus for providing a fast and accurate alternative to the golden standard of Monte‐Carlo‐based calculations, especially when inhomogeneous tissues are involved, resulted in the exploration of new strategies.

One such strategy is the application to external beam radiotherapy of a deterministic solution of the linear Boltzmann transport equation (LBTE). A benefit of the deterministic radiation transport solutions of the LBTE compared to Monte Carlo simulations is the lack of statistical noise in the calculated dose. An algorithm using this technique, born on the prototype solver called Attila,^5^ was first used in the radiotherapy environment for dose calculation in brachytherapy treatments giving accurate radiation transport solutions for implanted radioactive sources.^5^, ^6^

Based on this prototype, a dose calculation algorithm for external photon beams has been developed on the same methods and implemented in the Varian Eclipse external beam treatment planning system (Varian Medical Systems, Palo Alto, CA, USA). This new algorithm is the Acuros XB Advanced Dose Calculation algorithm (Acuros XB) and was first benchmarked by Fogliata et al.^7^ in water, and further validated by Han et al.^8^ on a Radiological Physics Center's head and neck phantom. Kan et al.^9^ evaluated the dosimetric impact of Acuros XB on intensity modulated stereotactic radiotherapy for locally persistent nasopharyngeal carcinoma, and showed that Acuros XB improved the dose calculation accuracy from 41% to 6% at the air/tissue interface when compared with AAA. By testing on the anthropomorphic phantom, the author showed that the measured doses matched those of the Acuros XB to within 3%, while AAA overestimated the doses by up to 10%. All of the studies show the advantage of Acuros XB compared with AAA in terms of accuracy. The prior studies warrant further validation and exploration of the advantages of using Acuros in clinics. Hence, the purpose of this study was to present implementation of Acuros XB for a Varian Clinac iX linac, and further validate Acuros XB by comparing it with measurements and the AAA algorithm using homogeneous and inhomogeneous phantoms. Dose distribution differences in VMAT plans on phantoms as well as on patients were also compared. The differences between doses calculated by AAA and Acuros dose‐to‐water and dose‐to‐medium on patient plans were also compared

Before presenting the methods and materials, it would be helpful to first give a short description of the algorithms used in this study. The two algorithms implemented in the Eclipse treatment planning system for all dose calculations in this study were the Acuros XB version 11 and the AAA version 11. The AAA is a kernel based convolution/superposition method and was originally developed to improve the dose calculation accuracy in heterogeneous media. The kernels, representing the energy transport and dose deposition of secondary particles stemming from a point irradiation, are not usually accessible through measurements but are very simple to calculate by use of Monte Carlo particle transport codes. The AAA corrects for heterogeneities by performing density scaling of Monte Carlo derived kernel for a homogeneous medium such as water. The common approach is to scale all dose fractions of a point kernel $h_{\rho 0}\left(s,r\right)$, calculated for a homogeneous medium of mass density $\rho_{0}$, by the mean electron density between the point *s* of energy release and the point *r* of energy deposition, that is,

$$h_{het}\left(s,r\right)=\frac{\rho \left(r\right)}{\rho 0}c^{2}h_{\rho 0}\left[c\left(r-s\right)\right]$$

where

$$c=c\left(s,r\right)=\int_{0}^{1}\rho_{rel}\left[s-l\left(s-r\right)\right]dl$$

in which $\rho_{rel}$ is the relative number of electrons per volume as compared with the reference medium (Tillikainen et al.^10^).

Similar to the Monte Carlo method, Acuros XB also belongs to one of the approaches of obtaining open form solution to the LBTE. In general, Monte Carlo method refers to the method in which random sampling of known probability distribution is used to solve a mathematical or physical problem. In the case of calculating dose, instead of directly solving the LBTE, Monte Carlo method indirectly obtains the solution by following the histories of a large number of particle transports through successive random samplings in media. The random sampling of known probability distribution function inevitably produces stochastic uncertainties when insufficient number of particle histories are followed. To achieve a certain level of accuracy, a huge amount of particle histories need to be sampled, therefore, Monte Carlo dose calculation is often too time‐consuming to be used. In contrary to Monte Carlo method, Acuros XB explicitly solves the LBTE by numerical methods. Acuros XB discretizes in space, angle and energy. For spatial discretization, the computational volume is subdivided into variable sized Cartesian elements, where material properties are assumed to be constant within each computational element. The discretization resolution in space, angle, and energy can produce systematic errors. However, with sufficient fine‐tuning, both methods should converge on the same solution (Fogliata et al.,^7^ Gifford et al^5^).

Same as Monte Carlo, Acuros XB also provides two options of dose reporting modes, that is, dose‐to‐water, D_w, m_, and dose‐to‐medium, D_m,m_. Both calculate the energy‐dependent electron fluence based on material properties of the interested media. The process for D_w,m_ and D_m,m_ is the same during the Acuros XB transport calculation. The difference between them is mainly in the post‐processing step, during which the energy‐dependent fluence resulted from transport calculation is multiplied by different flux‐to‐dose response functions to obtain the absorbed dose value. Acuros XB uses a medium‐based response function for D_m,m_ and a water‐based response function for D_w,m_. The absorbed dose to water D_w,m_ is related to the absorbed dose to medium by

$$D_{w,m}=D_{m,m}*S_{w,\mathrm{med}}$$

where S_w,med_ is the unrestricted water‐to‐medium mass collision stopping power ratio averaged over the energy spectra of primary electrons. In this work, Acuros_D_w,m_ was compared with measurements and dose calculated by AAA, and at the end, AAA dose were compared with both Acuros_D_w,m_ and Acuros_D_m,m_ on three VMAT plans for lung patients.

## 2 MATERIALS AND METHODS

### 2.A Beam data

The same set of beam data used by AAA version 11 measured by two IBA CC13 ion chambers (IBA, Barlett, TN) in a 3D Blue Phantom (Wellhöfer, IBA Dosimetry America, Barlett, TN, USA) for field sizes 2 × 2–40 × 40 cm^2^ were imported for the configuration of Acuros XB 11. All data used in this study were taken for 6 & 23 MV photon beams generated from a Varian Clinac iX accelerators equipped with a Millennium 120 multileaf collimator (Varian Medical Systems, Palo Alto, CA, USA). After commissioning the Acuros XB, measurements of the point dose were performed for field sizes ranging from 6 × 6 to 40 × 40 cm^2^ on the same water tank and the measurements were compared with dose calculated by AAA and Acuros.

The dose calculation grid resolution can be set by users from 1 to 5 mm for AAA and 1 to 3 mm for Acuros XB during the treatment planning. In this study, the dose grid size was set at 2.5 mm which is typically used in our clinics.

### 2.B Verification phantoms

All measurements on homogeneous phantom were carried out using a water‐proof CC13 ionization chamber (Wellhöfer, Nashville, TN) on an IBA Blue (Wellhöfer, IBA Dosimetry America, Bartlett, TN, USA) water tank with dimensions (LxWxH) 48 cm × 48 cm × 41 cm. The measurements were performed with 100 cm SSD and 200 MUs on a Varian iX machine, and the effective point of measurement was defined as 0.6*r*_*cav*_ (*r*_*cav*_ is the radius of the ion chamber) upstream from the central axis of the ion chamber.^11^ Points on and off central beam axis with different depths were measured for open beam sizes varying from 6 × 6 cm to 40 × 40 cm.

Table 1 summarizes the locations of measured points for open fields. These measurements were done for both 6 MV and 23 MV photon beams. Due to the memory limit of our TPS computer, all fields except 40 × 40 cm for 23 MV beam were calculated (Acuros XB and AAA) and compared with measurements.

**Table 1:** Locations of the measured points on a homogeneous water phantom for open beams. Point depths were 5, 10 and 20 cm for all field sizes

	Beam axis	+2.5 cm off beam axis	+5.0 cm off beam axis	+10 cm off beam axis	+15 cm off beam axis
6 cm × 6 cm point depth (cm)	5, 10 and 20				
10 cm × 10 cm point depth (cm)	5, 10 and 20	5, 10 and 20			
20 cm × 20 cm point depth (cm)	5, 10 and 20	—	5, 10 and 20		
30 cm × 30 cm point depth (cm)	5, 10 and 20	—	5, 10 and 20	5, 10 and 20	
40 cm × 40 cm point depth (cm)	5, 10 and 20	—	5, 10 and 20	5, 10 and 20	5, 10 and 20

Physical wedges are routinely used in our clinics and were commissioned in both Acuros XB and AAA. In this study, Acuros XB and AAA were compared and validated for 15, 30, 45 and 60° wedges, and a typical 15 cm × 15 cm field size was chosen for all the wedge angles. Similar to the open fields, the depth of the points were 5, 10, and 20 cm, respectively. At each depth, three points were measured, and two of the points are off‐axis points, where −5 cm means 5 cm towards the heel and +5 cm means 5 cm towards the toe from central axis of the beam.

A rectangular inhomogeneous phantom with a lung‐equivalent insert on one side was used to check the accuracy of Acuros XB in the presence of inhomogeneity for 6 MV and 23 MV beams. The phantom were irradiated on a Varian iX machine and six points located in and near the lung equivalent region were measured and compared with calculations by Acuros and AAA.

**Figure 1:**
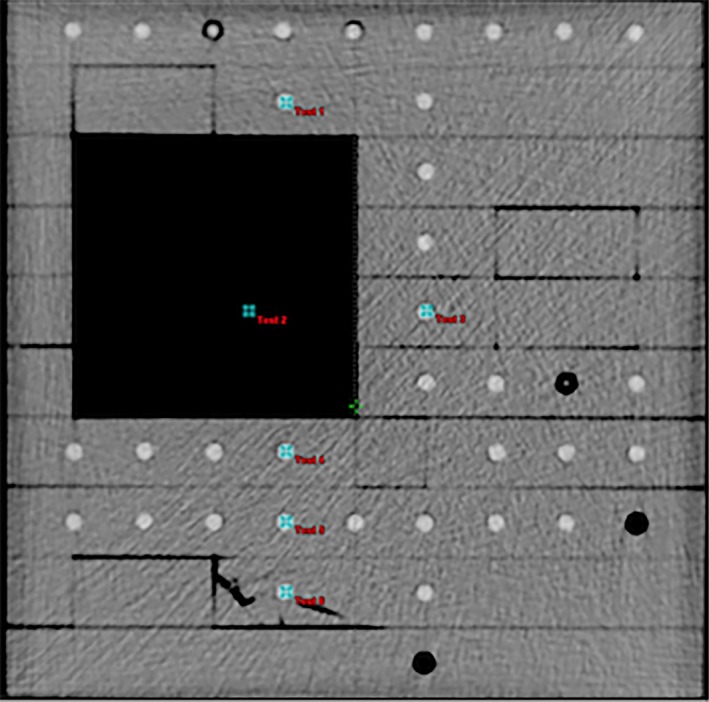
Inhomogeneous phantom used in this study. Six points were measured and the measurements were compared with calculations.

Figure 1 shows the axial view of the phantom with the locations of the six points marked as test 1, test 2, …, test 6. Exradin A1SL ionization chamber (standard imaging, Middleton, WI, USA) was used to measure the dose. The measurements were made for two symmetrical (8 × 8 cm^2^ and 15 × 15 cm^2^) fields and an asymmetrical (X1 = 8, X2 = 0, Y1 = 7.5 and Y2 = 7.5 cm) 8 × 15 cm^2^ field. Wedged fields were also checked and the 15 × 15 cm^2^ field size was chosen for all wedge angles.

**Figure 2:**
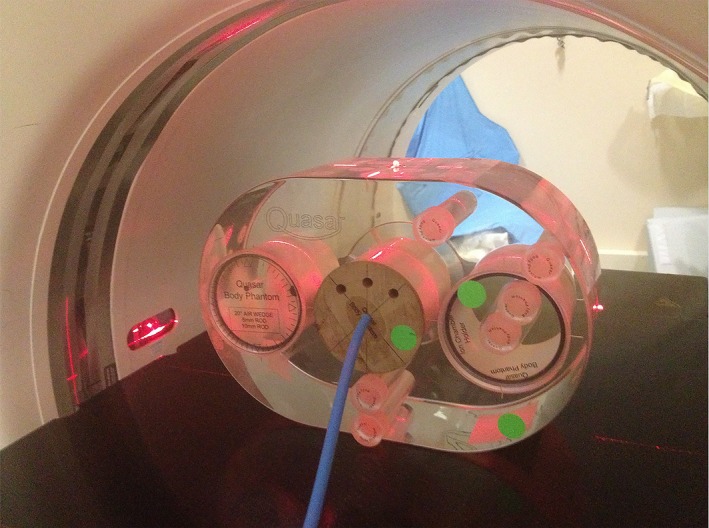
QUASAR phantom used to check the accuracy of dose calculation algorithms in lung equivalent material for clinically used VMAT plans.

In addition to the inhomogeneous phantom, Quality Assurance System for Advanced Radiotherapy (QUASAR, Modus Medical Devices Inc., London, ON, Canada) was used to check the accuracy of the Acuros XB and AAA in low density region for clinically used VMAT plans. These validations not only compared the dose calculation algorithms in lung equivalent material, they also examined the dose calculation accuracies in the presence of dynamic MLC movements. The QUASAR phantom consists of two main parts: a programmable drive unit and a body oval with lung equivalent (cedar) cylindrical insert. For the purpose of validating the dose calculation algorithms, the CT images of the body oval and lung insert with a PTW N31003 at the center were imported into our Treatment Planning System, and three clinically‐used VMAT plans for lung patients were calculated on this set of CT images. Figure 2 shows the setup of the phantom with the ionization chamber at the center. Three VMAT plans were delivered onto the phantom on our treatment machine (Varian iX), and the doses measured by the chamber were compared with calculated doses in Eclipse. The model N31003 (formerly N233641) has a nominal volume of 0.3 cc and an internal dimensions of 5.5 mm diameter and 16.25 mm length. A structure of the chamber with a 5.5 mm diameter and 16.25 mm length was created in Eclipse and its mean doses were compared with measurements.

Historically, radiotherapy dose measurements and calculations have been performed in, or specified in terms of the absorbed dose‐to‐water (D_w,m_). Like Monte Carlo dose calculation, Acuros provides two dose reporting mode dose‐to‐water (Acuros_D_w,m_) and dose‐to‐medium (Acuros_D_m,m_). Whether one should eventually use Acuros_D_m,m_ in place of Acuros_D_w,m_ in clinical prescriptions is an interesting research topic^12^, ^13^, ^14^. Since AAA has been used in clinics for years, hence, to make a correct transition from AAA to either Acuros_D_w,m_ or Acuros_D_m,m_, it is important to evaluate the differences between dose calculated by AAA and Acuros_D_w,m_ (and Acuros_D_m,m_). Therefore, three lung VMAT plans were chosen, and, for each of the VMAT plans, doses calculated by AAA and Acuros_D_w,m_ (Acuros_D_m,m_) were compared. The three plans has been approved and used in our clinics. Dose at the isocenter, maximum cord dose and D_100_ of GTV (the minimum dose in the GTV) were compared. In addition, DVHs of GTV calculated by AAA and Acuros_D_w,m_ (Acuros_D_m,m_) were compared and the dose distributions were shown side‐by‐side for visual comparison.

## 3 RESULTS

### 3.A Results on homogeneous phantom

Measured and calculated (AAA and Acuros XB) doses for 6X photon beams of variable field sizes are reported in Table 2. Relative standard errors (RSE) which is defined as (std/mean) of measurements * 100 are also listed in the table. The difference was calculated as follows

(CALCULATED DOSE‐MEASURED DOSE)/MEASURED DOSE∗100

**Table 2:** Percentage dose differences between calculations (Acuros_D_w,m_ and AAA) and measurements for 6MV photon beams on homogeneous phantom

	Beam axis			2.5 cm off beam axis			5.0 cm off beam axis			10 cm off beam axis			15 cm off beam axis		
	%DIFF		RSE %	%DIFF		RSE %	%DIFF		RSE %	%DIFF		RSE %	%DIFF		RSE %
	Acuros	AAA		Acuros	AAA		Acuros	AAA		Acuros	AAA		Acuros	AAA	
6 × 6 cm															
d = 5 cm	0.2	−0.3	0.084												
d = 10 cm	0.4	−0.1	0.081												
d = 20 cm	0.8	1.1	0.079												
10 × 10 cm															
d = 5 cm	0.1	−0.4	0.081	0.3	0	0.07									
d = 10 cm	0.2	−0.1	0.08	0.4	0.1	0.071									
d = 20 cm	0.2	1.4	0.079	0.3	1.3	0.069									
20 × 20 cm															
d = 5 cm	−0.2	−0.8	0.078				−0.2	−0.7	0.075						
d = 10 cm	−0.1	−0.5	0.076				−0.1	−0.6	0.073						
d = 20 cm	0.4	0.1	0.073				0.1	−0.1	0.071						
30 × 30 cm															
d = 5 cm	0	−0.7	0.082				0.1	−0.6	0.077	−0.1	−0.6	0.077			
d = 10 cm	0.2	−0.5	0.08				0.2	−0.6	0.075	0.2	−0.4	0.075			
d = 20 cm	0.4	−0.4	0.08				0.4	−0.3	0.075	0.4	−0.1	0.074			
40 × 40 cm															
d = 5 cm	0.2	−0.8	0.081				0.1	−0.8		0.1	−0.5	0.076	−0.2	−0.7	0.081
d = 10 cm	0.5	−0.7	0.080				0.4	−0.9		0.5	−0.5	0.076	0.2	−0.8	0.08
d = 20 cm	0.8	−0.7	0.078				0.7	−0.8		0.8	−0.5	0.072	0.7	−0.5	0.08

The accuracy of AAA was comparable to that of Acuros XB. In general, Acuros XB calculations were within 1% of measured dose. For most field sizes and depths, AAA calculations were lower than measured dose except at 20 cm depth for field sizes 6 × 6, 10 × 10 and 20 × 20. Similar to Table 2, measured and calculated (AAA and Acuros XB) doses for 23 MV photon beam are listed in Table 3. Generally, Acuros XB achieved equivalent accuracies as AAA for both 6 and 23 MV photon beams on homogeneous phantom.

**Table 3:** Percentage dose differences between calculations (Acuros_D_w,m_ and AAA) and measurements for 23 MV photon beams on homogeneous phantom

	Beam axis			2.5 cm off beam axis			5.0 cm off beam axis			10 cm off beam axis		
	%DIFF		RSE %	%DIFF		RSE %	%DIFF		RSE %	%DIFF		RSE %
	Acuros	AAA		Acuros	AAA		Acuros	AAA		Acuros	AAA	
6 × 6 cm												
d = 5 cm	−0.3	−0.2	0.047									
d = 10 cm	0	−0.4	0.042									
d = 20 cm	0	0	0.042									
10 × 10 cm												
d = 5 cm	−0.8	−1.5	0.043	−1.2	−2	0.042						
d = 10 cm	−0.3	−0.9	0.042	−0.9	−1.6	0.041						
d = 20 cm	−0.4	−0.7	0.042	−0.9	−1.3	0.041						
20 × 20 cm												
d = 5 cm	−0.8	−1.3	0.042				−1.5	−2.0	0.043			
d = 10 cm	−0.5	−1.0	0.041				−1.3	−1.7	0.041			
d = 20 cm	−0.3	−0.9	0.04				−1.3	−1.7	0.041			
30 × 30 cm												
d = 5 cm	−0.5	−1.0	0.04				−1.2	−1.9	0.041	−1.2	−1.5	0.042
d = 10 cm	−0.4	−1.0	0.039				−1.0	−1.7	0.04	−0.9	−1.5	0.039
d = 20 cm	−0.4	−1.0	0.039				−1.2	−1.7	0.039	−1.0	−1.3	0.04

The accuracy of wedged fields for 6 MV and 23 MV photon beams are reported in Tables 4 and 5, respectively. Consistent with what we observed for open fields, Acuros XB improved or achieved equivalent accuracy for wedged fields when compared with AAA. For both Acuros XB and AAA, the differences between calculations and measurements were within 3% and the differences were larger for higher energy beams.

**Table 4:** Percentage dose differences between calculations (Acuros_D_w,m_ and AAA) and measurements for 6 MV wedged fields. The wedged field size was set to 15 × 15 cm

	−5.0 cm off beam axis			Beam axis			+5.0 cm off beam axis		
	%DIFF		RSE %	%DIFF		RSE %	%DIFF		RSE %
	Acuros	AAA		Acuros	AAA		Acuros	AAA	
15°									
d = 5 cm	0	−0.8	0.073	−0.3	−0.8	0.068	0.3	−1.1	0.07
d = 10 cm	−0.3	−1.1	0.075	−0.4	−0.6	0.067	0.1	−1.1	0.068
d = 20 cm	−0.3	−0.5	0.072	−0.3	0.6	0.068	−0.1	−0.4	0.067
30°									
d = 5 cm	0.3	−0.4	0.076	−0.2	−1.1	0.067	1.1	−0.3	0.073
d = 10 cm	0.1	−0.4	0.075	−0.1	−0.9	0.066	0.7	−0.4	0.072
d = 20 cm	0	−0.2	0.071	0.0	0.2	0.065	0.4	−0.4	0.07
45°									
d = 5 cm	0.1	−0.3	0.077	−0.2	−0.8	0.07	0.3	−0.2	0.07
d = 10 cm	0.0	−0.7	0.075	−0.2	−0.8	0.07	0.1	−0.6	0.068
d = 20 cm	−0.1	−0.1	0.075	−0.3	−0.1	0.069	−0.1	−0.3	0.067
60°									
d = 5 cm	−1.1	−1.1	0.074	−0.4	−1.5	0.071	−0.7	−1.2	0.072
d = 10 cm	−1.0	−1.3	0.074	−0.4	−1.3	0.069	−0.9	−1.8	0.07
d = 20 cm	−0.8	−1.2	0.073	−0.3	−0.6	0.066	−1.1	−1.5	0.07

**Table 5:** Percentage differences between calculations (Acuros_D_w,m_ and AAA) and measurements for 23 MV wedged fields

	−5.0 cm off beam axis			Beam axis			+5.0 cm off beam axis		
	%DIFF		RSE %	%DIFF		RSE %	%DIFF		RSE %
	Acuros	AAA		Acuros	AAA		Acuros	AAA	
15°									
d = 5 cm	0.1	−0.5	0.05	−1.2	−0.9	0.049	−1.0	−2.0	0.046
d = 10 cm	−0.2	−1.0	0.048	−1.3	−1.6	0.047	−1.3	−2.6	0.045
d = 20 cm	−0.4	−0.5	0.047	−1.7	−1.4	0.043	−1.7	−2.3	0.045
30°									
d = 5 cm	0.5	−0.7	0.048	−1.5	−1.3	0.048	−0.5	−2.1	0.048
d = 10 cm	0.4	−1.2	0.043	−1.2	−2.0	0.043	−0.6	−2.7	0.046
d = 20 cm	0.7	−0.7	0.043	−0.8	−1.6	0.042	−0.5	−2.3	0.046
45°									
d = 5 cm	−0.3	−0.8	0.046	−1.3	−1.5	0.044	−1.2	−2.1	0.047
d = 10 cm	−0.1	−1.6	0.045	−1.0	−2.2	0.042	−1.0	−2.9	0.047
d = 20 cm	0.8	−0.9	0.043	−0.2	−1.8	0.042	−0.5	−2.7	0.045
60°									
d = 5 cm	−1.3	−0.9	0.048	−1.7	−1.6	0.048	−2.5	−2.6	0.046
d = 10 cm	−1.2	−1.2	0.045	−1.4	−2.0	0.045	−2.4	−2.8	0.044
d = 20 cm	−0.1	−0.4	0.044	−0.5	−1.2	0.044	−1.8	−2.1	0.044

### 3.B Results on inhomogeneous phantom

Tables 6 and 7 present percentage differences between calculated (AAA and Acuros XB) and measured doses on the inhomogeneous phantom. “test 3” was not measured for the asymmetrical field (8 × 15) since it is outside the field. Also “test 5” was not measured for wedged fields since it is very similar to “test 4” and “test 6”. Compared with AAA, Acuros XB significantly improved the dose calculation accuracy for the points located beyond (downstream) the inhomogeneous region, that is, points “test 4”, “test 5” and “test 6”. For most of them, doses calculated by Acuros XB were within 3% of measurements while the maximum difference between measurement and calculation by AAA was more than 8%.

**Table 6:** Percentage dose difference between calculations and measurements for 6 MV photon beams on the inhomogeneous phantom. Acuros_D_w,m_ and AAA were compared side‐by‐side for various open and wedged fields

	Test 1		Test 2		Test 3		Test 4		Test 5		Test 6	
	Acuros	AAA	Acuros	AAA	Acuros	AAA	Acuros	AAA	Acuros	AAA	Acuros	AAA
8 × 8 open	−0.5	−0.15	1.06	1.5	1.06	−0.82	0.87	7.91	1.48	7.5	1.26	6.44
10 × 10 open	−0.88	−0.28	−0.33	2.36	0.22	−1.52	0.51	7.96	1.16	7.7	0.96	6.96
8 × 15 ofst	−0.76	0.02	1.09	0.01	−	−	0.87	8.68	1.64	8.96	1.66	8.52
15 × 15 op	−1.11	−0.69	1.04	1.7	0.13	−2.07	0.24	7.1	0.84	7.5	0.58	7.18
15 × 15 w15	−0.78	−0.14	1.44	1.44	0.19	−1.71	−0.05	6.47	−	−	0.05	6.51
15 × 15 w30	−0.67	−3.2	1.37	1.06	−0.03	−1.84	0.1	6.58	−	−	0.05	6.31
15 × 15 w45	−0.86	1.23	1.98	1.32	−0.35	−1.26	0.07	7.12	−	−	−0.77	7.06
15 × 15 w60	−0.86	1.62	1.98	1.16	−0.35	−1.12	0.07	7.86	−	−	−0.77	7.54

**Table 7:** Percentage dose difference between calculated (Acuros_D_w,m_ and AAA) and measured dose for 23 MV photon beams on inhomogeneous phantom

	Test 1		Test 2		Test 3		Test 4		Test 5		Test 6	
	Acuros	AAA	Acuros	AAA	Acuros	AAA	Acuros	AAA	Acuros	AAA	Acuros	AAA
8 × 8 open	−1.78	−0.9	1.65	−1.64	−0.3	0.43	−2.46	0.48	−0.59	3.46	−0.54	3.56
10 × 10 open	−2.07	−0.58	0.62	−1.49	−0.31	0.59	−1.42	1.27	−0.37	3.83	−0.5	4.07
8 × 15 ofst	−2.16	−0.43	−1.77	−2.96	−	−	−1.35	1.46	0.18	4.31	0.24	4.67
15 × 15 op	−2.5	−0.8	−0.62	−1.7	−0.65	−0.02	−1.52	1.5	−0.62	3.5	−0.88	3.72
15 × 15 w15	−1.44	−0.92	−0.15	−2.59	−0.19	−0.55	−1.36	0.92	−	−	−1.02	2.76
15 × 15 w30	0.16	1.51	1.27	−0.43	0.64	1.54	−0.25	2.88	−	−	0.44	5.19
15 × 15 w45	−0.24	2.24	2.08	0.26	0.44	1.68	0.59	4.13	−	−	0.91	6.44
15 × 15 w60	−0.89	−0.22	0.4	−0.5	0.33	1.71	0.44	4.32	−	−	0.59	6.71

### 3.C Results on quasar phantom and patient anatomy

**Figure 3:**
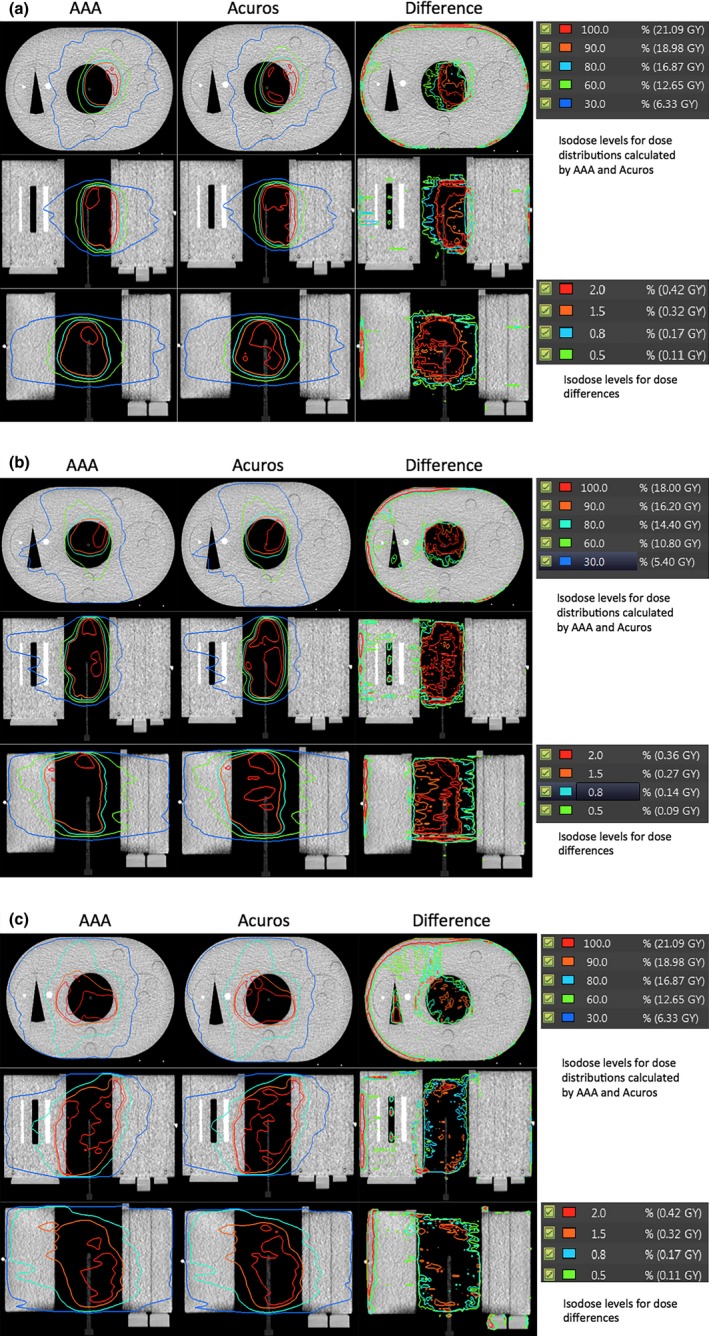
Dose distributions calculated by AAA and Acuros_D_w,m_ on QUASAR phantom and their differences in three views. The differences were calculated via: Acuros_D_w,m_ — AAA dose. The percentage differences were relative to the dose calculated at iso‐center for 10 fractions.

Table 8 presents the percentage differences between measurements and calculations at the center of the lung equivalent insert of the QUASAR phantom. Acuros calculations were within 1% of measurements while the maximum difference between calculation by AAA and measurement was 2.7%. Doses calculated by Acuros were either higher or lower than measured doses, however, AAA underestimated doses in all three cases. The dose profiles calculated by Acuros and AAA and their differences (Acuros – AAA) on the QUASAR phantom are shown side‐by‐side in Fig. 3. Most of the differences between AAA and Acuros XB were between 1 and 2.5 percent and they were mainly in low density region. Also when calculating the differences, dose calculated by AAA was subtracted from the dose calculated by Acuros XB (Acuros XB dose — AAA dose). The differences were mostly in the lung insert region, and this implied that dose calculated by Acuros XB was higher than the dose calculated by AAA in this region and it is consistent with what we observed in Table 8.

**Table 8:** Percentage differences between measured and calculated dose at the center of lung equivalent insert of the QUASAR phantom

	Measured dose (cGy)	Dose calculated by Acuros *D*_*w,m*_ (cGy)	Percent difference for Acuros *D*_*w,m*_	Dose calculated by AAA (cGy)	Percent difference for AAA
Patient 1	209.6	210.4	0.38	206.5	−1.5
Patient 2	180.5	179.0	−0.83	175.6	−2.7
Patient 3	211.1	211.2	0.06	208.4	−1.3

**Figure 4:**
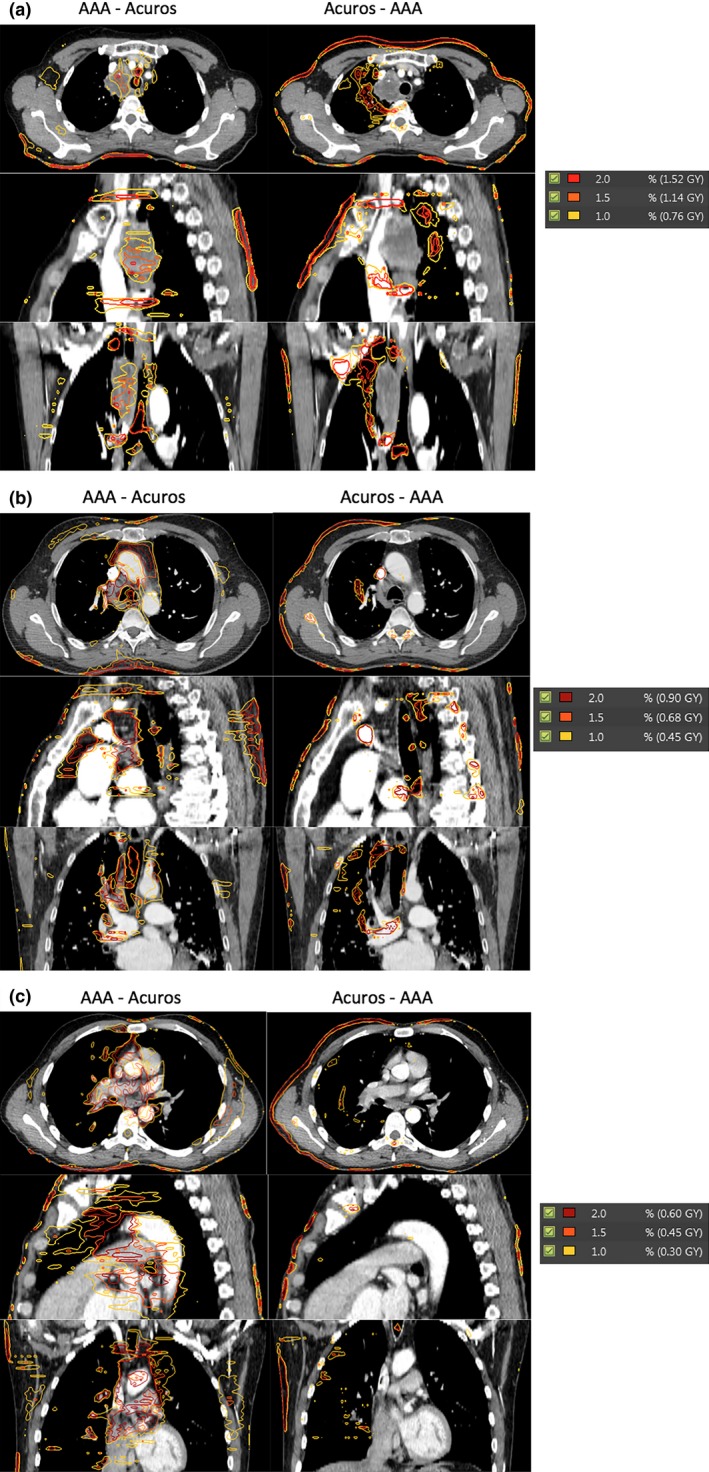
Dose differences are shown for three patients. Instead of absolute differences, two dose distributions (AAA dose — Acuros D_w,m_ and Acuros D_w,m_ — AAA dose) are shown side‐by‐side in (a), (b) and (c).

Figure 4 shows the dose differences (calculated by AAA and Acuros_D_w,m_) side‐by‐side on the patient anatomy for three VMAT patients. To illustrate the regions where dose calculated by Acuros is higher (or lower) than that calculated by AAA, two dose distributions, AAA dose — Acuros dose and Acuros dose — AAA dose, are shown side‐by‐side. The spatial distributions implied that dose calculated by AAA was lower in low density lung equivalent tissue while it was higher in normal soft tissue when compared with the dose calculated by Acuros. The prescriptions for the three plans were 76, 45 and 30 Gy, respectively.

Table 9 shows percentage differences between doses calculated by AAA and doses calculated by Acuros (Acuros_D_w,m_ and Acuros_D_m,m_, respectively) at the isocenter. The isocenters were all located inside the PTV. The differences between doses calculated by AAA and Acuros_D_w,m_ were less than 2%, but the maximum differences between AAA dose and Acuros_D_m,m_ was 4.3%. Similar to Table 9, Table 10 shows percentage differences between maximum spinal cord dose calculated by AAA and Acuros_D_w,m_ (Acuros_D_m,m_). In general the differences between AAA dose and Acuros_D_w,m_ were less than or equal to 1%, but the differences between AAA dose and Acuros_D_m,m_ for the spinal cord were more than 3%.

**Table 9:** Percentage differences between doses calculated by AAA and Acuros (D_w,m_ and D_m,m_, respectively) at the isocenter

	AAA dose at isocenter (cGy)	Acuros_D_w,m_ at isocenter(cGy)	Percent difference for Acuros_D_w,m_	Acuros_D_m,m_ at isocenter(cGy)	Percent difference for Acuros_D_m,m_
Patient 1	205.9	205.9	0	209.0	1.5
Patient 2	189.5	186.5	−1.58	182.2	−3.8
Patient 3	211.0	207.0	−1.89	202	−4.3

**Table 10:** Percentage differences between the maximum cord doses calculated by AAA and Acuros (D_w,m_ and D_m,m_, respectively)

	Maximum cord dose by AAA (cGy)	Maximum cord dose Acuros_D_w,m_ (cGy)	Percent difference for Acuros_D_w,m_	Maximum cord dose acuros_D_m,m_ (cGy)	Percent difference for acuros_D_m,m_
Patient 1	4647.0	4600.0	−1.0	4457.0	−4.1
Patient 2	2650	2640	−0.37	2550	−3.7
Patient 3	2070	2054	−0.77	2000	−3.4

**Figure 5:**
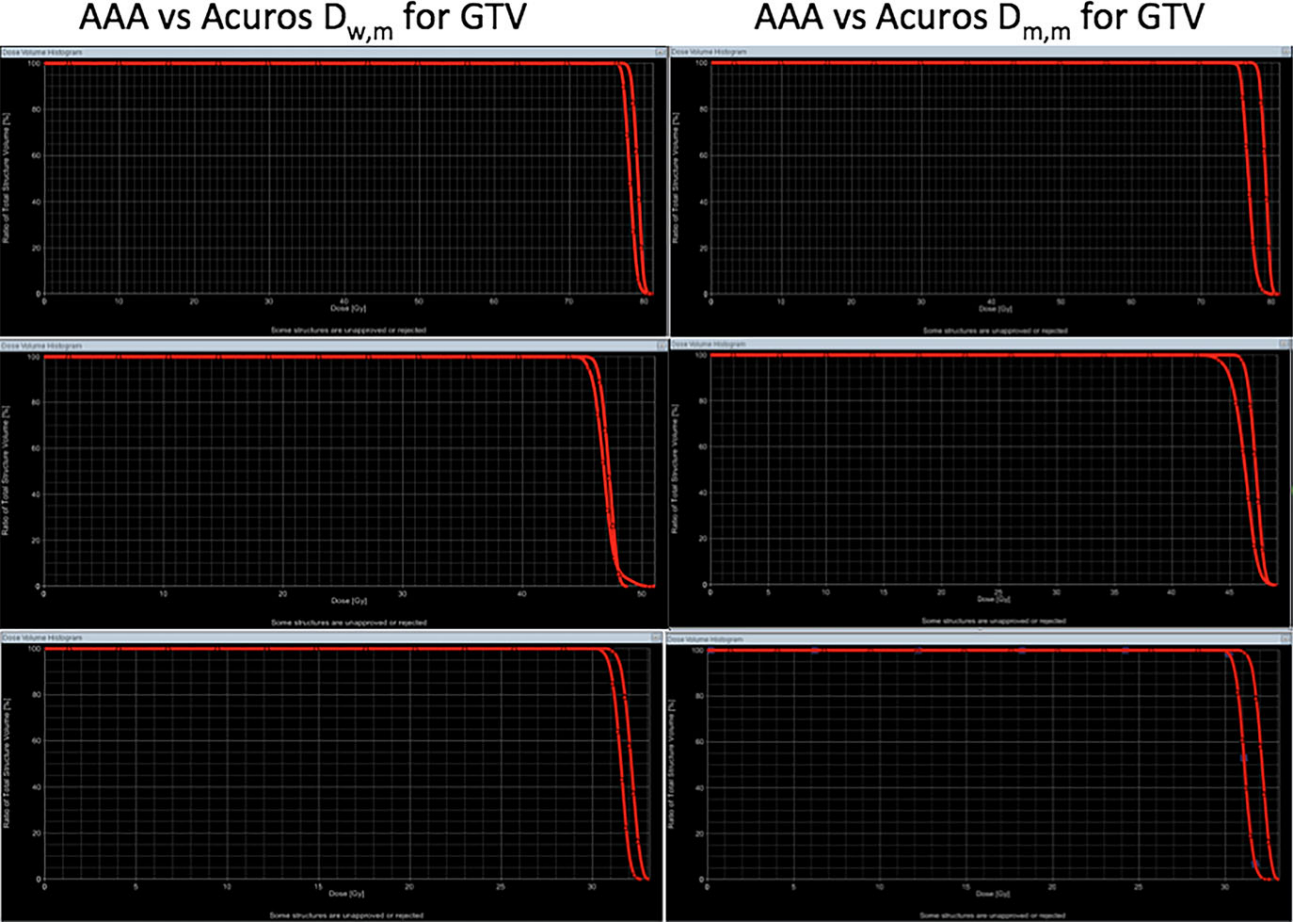
DVH comparison between AAA and Acuros (Acuros_D_w,m_ and Acuros_D_m,m_) for GTV. The left column shows the DVHs calculated by AAA and Acuros_D_w,m_, and right column shows the DVHs calculated by AAA and Acuros_D_m,m_. DVHs calculated by AAA are shown in triangles.

**Figure 6:**
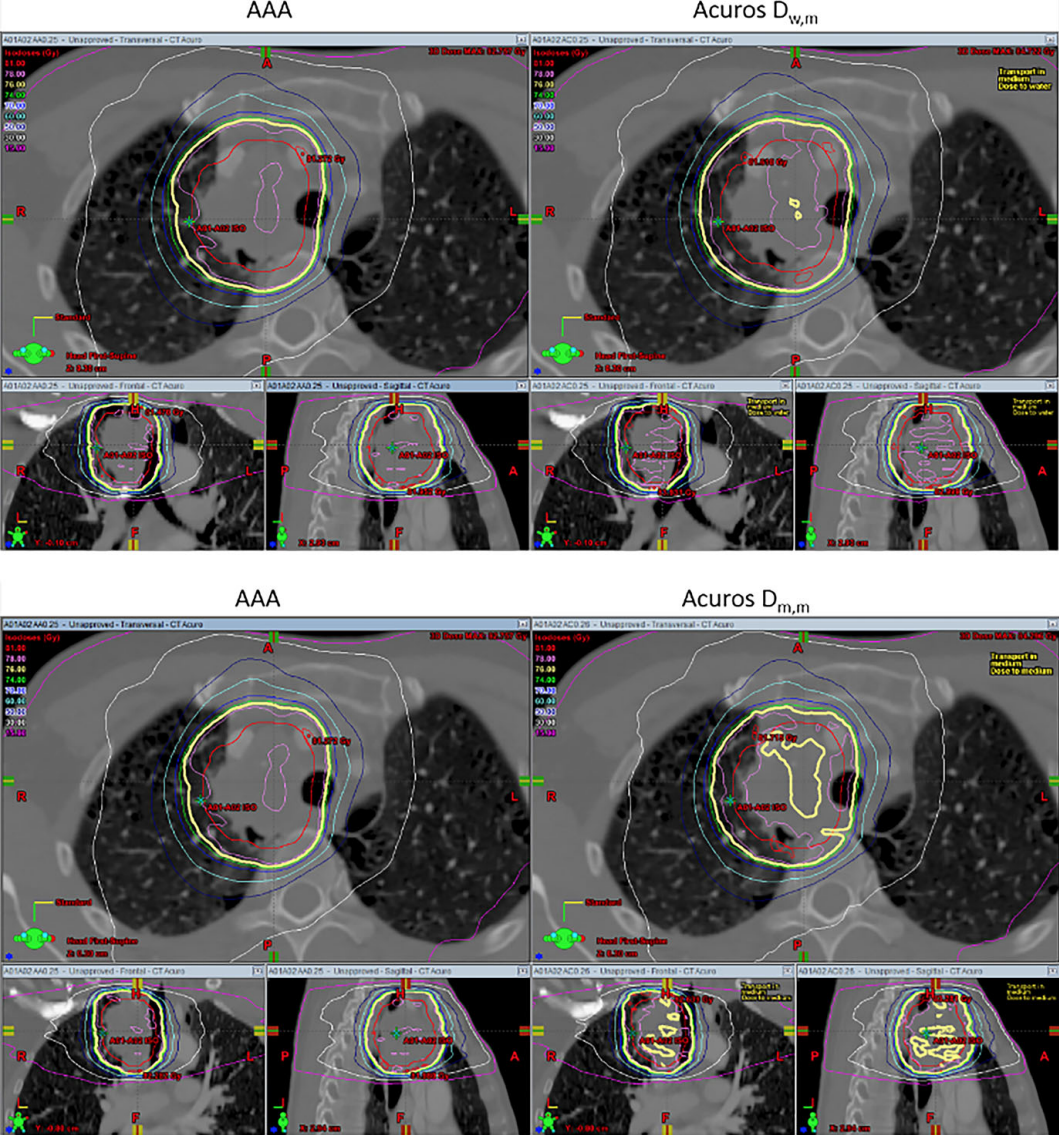
Top row compares the AAA dose with Acuros_D_w,m_, and the bottom row shows AAA dose and Acuros D_m,m_ side‐by‐side. The solid red line is the GTV and the thick yellow line is the prescribed dose (76 Gy).

**Figure 7:**
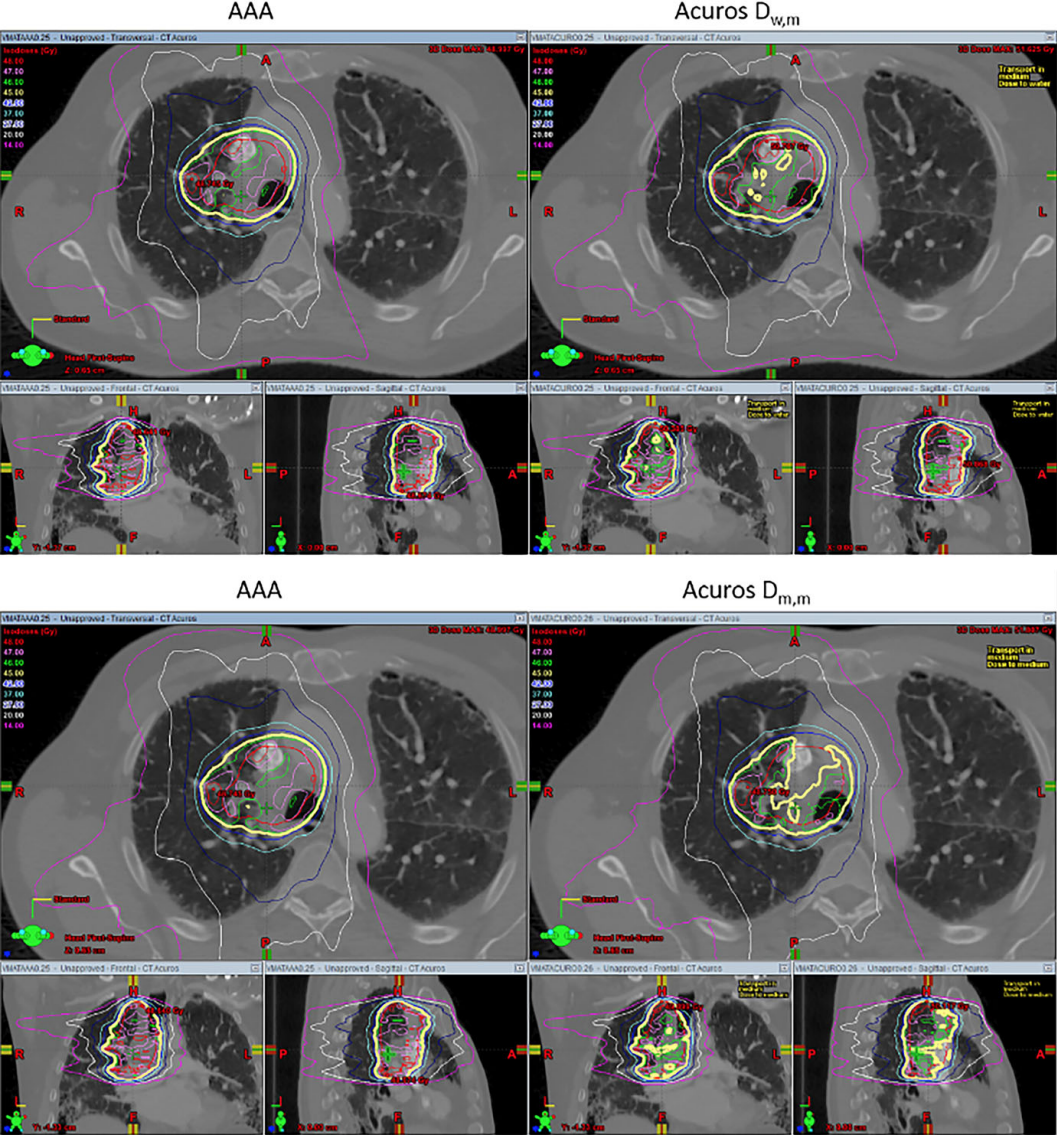
Similar to Fig. 6 but for patient 2. The prescribed dose is 45 Gy(thick yellow line). The GTV is shown in red.

**Figure 8:**
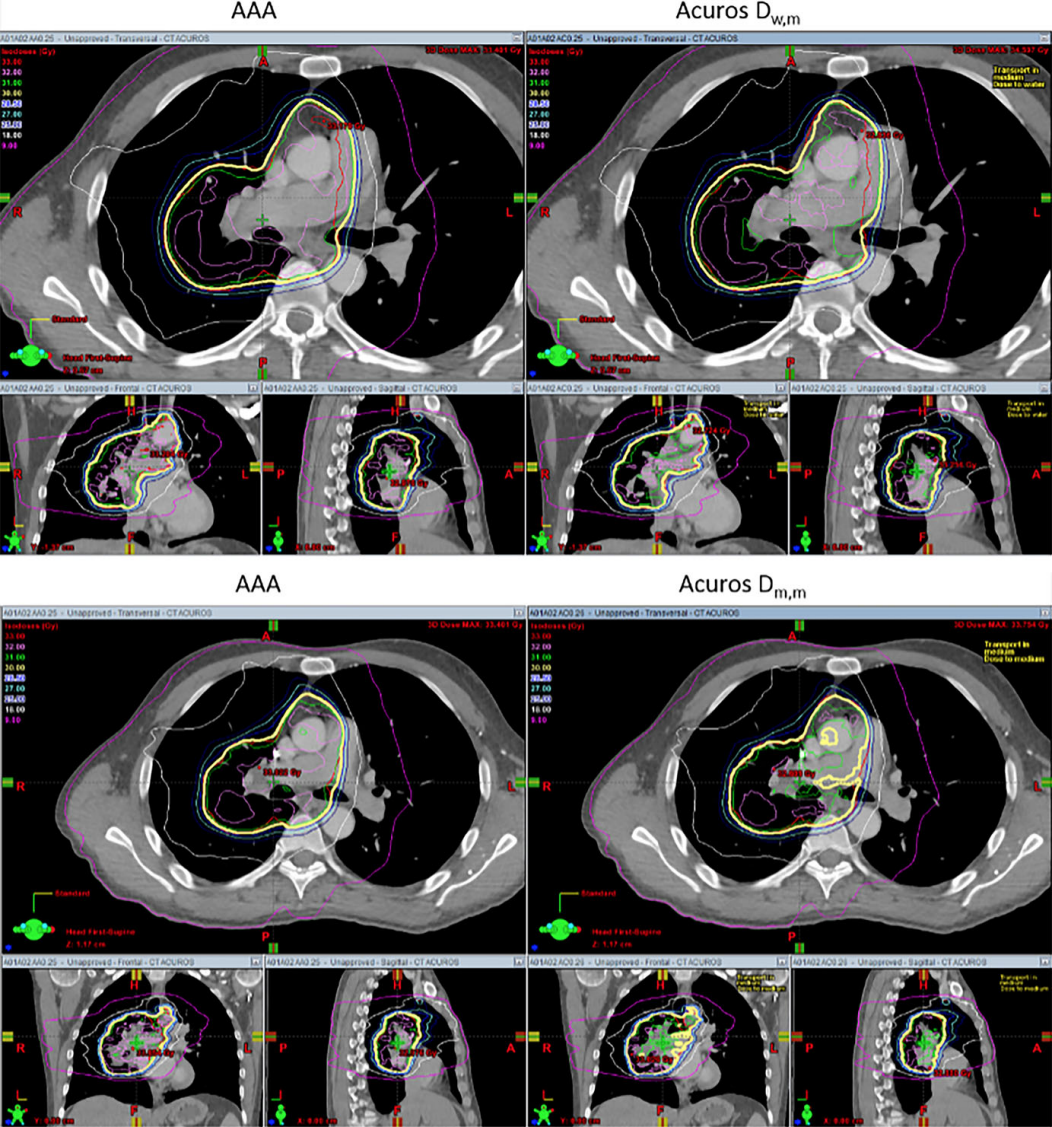
Comparison of dose distributions between AAA and Acuros_D_w,m_ (Acuros_D_m,m_). Prescribed dose is 30 Gy and the PTV is shown in red.

Figure 5 compares the DVHs' differences for GTVs of the three lung patients. For all three plans, the differences between AAA and Acuros_D_w,m_ is smaller than the differences between AAA and Acuros_D_m,m_. Another dosimetric criterion often used to evaluate the plan quality is D_100_ of GTV, and normally, D_100_ should be no less than prescribed dose in clinics. Table 11 shows the D_100_ calculated by AAA, Acuros_D_w,m_ and Acuros_D_m,m_ for the same plan. The differences in D_100_ between AAA and Acuros_D_w,m_ were less than 2%, but the differences between AAA and Acuros_D_m,m_ were more than 3% and the maximum difference was 11.3%. Dose distributions calculated by AAA and Acuros_D_w,m_ (Acuros_D_m,m_) along with GTVs (PTV for patient 3) were shown side‐by‐side in Figs. 6, 7, 8. By visual inspection, the differences between AAA and Acuros_D_m,m_ was larger than the differences between AAA and Acuros_D_w,m_.

**Table 11:** *D*_*100*_ of GTV calculated by AAA, Acuros_D_w,m_ and Acuros_D_m,m_ for three VMAT plans

	D_100_ of GTV by AAA (Gy)	D_100_ of GTV by Acuros_D_w,m_ (Gy)	Percent difference for Acuros_D_w,m_	D_100_ of GTV by Acuros_D_m,m_ (Gy)	Percent difference for Acuros_D_m,m_
Patient 1 (prescribed dose 76 Gy)	77.0	75.8	−1.5	73.5	−4.5
Patient 2 (prescribed dose 45 Gy)	45.0	44.1	−2.0	39.9	−11.3
Patient 3 (prescribed dose 30 Gy)	30.7	30.1	−1.9	29.6	−3.6

## 4 DISCUSSION

In this work, the implementation of a new photon dose calculation algorithm, Acuros XB, was described and its accuracy was validated against measurements and compared with our clinically used AAA algorithm. The validation was performed on both homogeneous and inhomogeneous phantoms for various open and wedged fields, the method described in our work is not limited to Acuros XB and can be used to validate any dose calculation algorithm before its clinic use. In addition, QUASAR phantom and clinically used VMAT plans were used to check the accuracy of Acuros XB and AAA in the presence of dynamic MLC movements and inhomogeneity. Since both Acuros XB and AAA use the same set of measured data to derive the source model, the dose calculation discrepancies between the two algorithms should come from the fundamental differences between the two algorithms in calculating the dose. When compared with traditional dose calculation algorithm (such as pencil), dose calculated by Monte‐Carlo or Acuros tends to be lower since Monte‐Carlo (Acuros XB) can account the loss of scatter better than traditional algorithms.^5^, ^6^ Interestingly, our study shows dose calculated by AAA was systematically lower than dose calculated by Acuros XB in lung equivalent region (Figs. 3, 4 and Table 8), this implies that AAA (version 11) might have over corrected the dose due to lack of scattering when compared with Acuros XB.

The results presented in this paper show that Acuros XB can achieve better or equivalent accuracy in homogeneous phantom while it significantly improves accuracy for single beam in inhomogeneous phantom as shown in Tables 6 and 7. For clinically used VMAT plans, Acuros XB also achieved better accuracy in low density tissue than AAA which underestimated doses for all three tested cases. Acuros XB and AAA calculations were also compared on patient CTs, and the differences indicate that AAA calculations were lower in lung region and higher in normal tissue (Fig. 4) when compared with Acuros XB which was consistent with the results that Acuros XB calculated doses were higher than AAA's calculations in low density region on the QUARSAR phantom.

For VMAT plans, Acuros XB is about three times faster than AAA. However, calculations of a single or few fields are longer with Acuros XB than AAA. For a clinically used 2‐fields spine plan, AAA calculated the dose in about 20 s while Acuros XB took 160 s on a Dell T5500 (with dual quad‐core Xeon 2.4 GHz processors and 24 GB memory).

AAA inherently only reports dose‐to‐water, but similar to Monte Carlo dose calculation algorithm, Acuros reports both dose‐to‐water and dose‐to‐medium. Previous publications^12^, ^13^, ^14^ recommend that when MC or grid‐based Boltzmann solver such as Acuros are used, dose‐to‐medium or Acuros_D_m,m_ computed inherently by these algorithms should be reported. From our comparison, we feel that further guidance and clarification is needed to make the transition from AAA to Acuros consistent and correct. Table 9 shows that the dose at isocenter can differ by up to 4.3% (patient 3) between AAA and Acuros_D_m,m_, and this could be significant in clinics since some physicians like to prescribe dose to a point such as the isocenter. If the same prescribed dose is used, it could potentially cause the MU differ by 4.3% between the plan calculated by AAA and the one calculated by Acuros_D_m,m_. As shown in Table 10, the maximum cord dose difference between AAA and Acuros_D_m,m_ is around 3%, and, more importantly, the difference looks like systematic difference (Acuros_D_m,m_ are lower than AAA dose for all three cases). Additional guidance should be given to physicists about how to handle the difference if it turned out to be systematic. However, it is worth mentioning that this study only compared VMAT plans for lung patients, and additional investigation is needed to confirm that maximum cord dose systematically differ by 3% between AAA and Acuros_D_m,m_. D_100_ of GTV is another dose criterion often used in clinics to evaluate the quality of a plan. Generally, D_100_ of GTV should be no less than prescribed dose. Table 11 shows that D_100_ calculated by AAA for the three VMAT plans all satisfied this criterion, but when the same plans were calculated by Acuros_D_m,m_, all three plans failed. For patient 2, the difference of D_100_ was 11.3% between the plan calculated by AAA and the plan calculated by Acuros_D_m,m_. If Acuros_D_m,m_ was used, then the plans for patient 1 and 2 should be rejected and plan for patient 3 was on the borderline. By comparing the DVHs (Fig. 5) and visually inspecting the dose distributions of AAA, Acuros_D_w,m_ and Acuros_D_m,m_ (Figs. 6, 7, 8), one can see that the coverage of GTV (PTV for patient 3) became a little bit worse if Acuros_D_w,m_ was used and much worse when Acuros_D_m,m_ was used.

Gladstone^14^, etc. recommends that, for MC or grid‐based Boltzmann solver (GBBS) algorithms such as Acuros XB, conversion of D_m,m_ to D_w,m_ should be avoided, rather, D_m,m_ computed inherently by these algorithms should be reported. Currently, our cancer center is participating NRG clinical trials and our AAA algorithm has been validated by Radiological Physics Center (RPC) and is used in clinics. Due to the large inconsistency between AAA and Acuros_D_m,m_, we decided to continue to use AAA until the root cause of the discrepancies are found. As a first step, we collaborated with a physicists from VARIAN and performed preliminary examinations, and tentatively concluded that the discrepancies were due to different type of tissue present in lung patients. However, to completely confirm this, more extensive tests are needed. To make such tests more robust and thorough, it would be better for us to collaborate with other institutions and VARIAN to perform such study with the goal of understanding whether the discrepancies seen in this study are inherent to Acuros XB and Monte Carlo. One particular test would be to commission Monte Carlo on our Clinic iX machine and use it to calculate the three plans and see whether Monte Carlo will report similar differences between AAA dose and dose‐to‐medium calculated by Monte Carlo. We leave these studies to our future work.

## 5 CONCLUSION

In general, the accuracy of Acuros XB photon dose calculation algorithm was found to be equivalent to that of AAA on homogeneous phantom and better than AAA on inhomogeneous phantom. It also achieves better agreement with measurements than AAA for clinically used VMAT plans in low density region on the QUASAR phantom. By comparing the point doses, DVHs of GTV and dose distributions calculated by AAA, Acuros_D_w,m_ and Acuros_D_m,m_, we can conclude that the differences between AAA dose and Acuros_D_w,m_ is smaller than the differences between AAA dose and Acuros_D_m,m_. In some cases, the differences are clinically significant, and hence, further clarification and guidance is needed before switching from AAA to Acuros_D_m,m_ in clinics.

## ACKNOLEDGMENTS

The authors would like to thank VARIAN physicists for their valuable support and discussion.

## CONFLICT OF INTEREST

The authors declare no conflict of interest.
